# Pretemporal transcavernous transtentorial approach for left pontine glioma

**DOI:** 10.3171/2019.10.FocusVid.19422

**Published:** 2019-10-01

**Authors:** Chih-Hsiang Liao, Chun-Fu Lin, Wei-Hsin Wang, Jui-To Wang, Shao-Ching Chen, Sanford P. C. Hsu

**Affiliations:** 1 Department of Neurosurgery, Neurological Institute, Taichung Veterans General Hospital, Taichung;; 2 Institute of Medicine, Chung Shan Medical University, Taichung;; 3 Division of General Neurosurgery, Neurological Institute, Taipei Veterans General Hospital, Taipei; and; 4 School of Medicine, National Yang Ming University, Taipei, Taiwan

**Keywords:** Meckel’s cave, pontine glioma, pretemporal, transcavernous, transtentorial, video

## Abstract

A 39-year-old man, who had a history of spinal myxopapillary ependymoma with cerebrospinal seeding status post twice operations and radiation therapy, presented with aggravating headaches, diplopia, dysphagia, and unsteady gait for 2 weeks. The brain MRI revealed a parenchymal lesion at the left aspect of the pons, about 2.8 × 2.3 × 3.2 cm^3^. The patient underwent a pretemporal transcavernous transtentorial approach for tumor removal. The pathological report showed an anaplastic astrocytoma. In this approach, a wider surgical corridor was obtained by opening the Meckel’s cave and cutting the tentorium, via which a safe entry point into the pons could be determined with neuromonitoring. In the authors’ opinion, this approach is safe and effective in selected ventrolateral pontine gliomas.

The video can be found here: https://youtu.be/sUt-9QFGgCI.

## Transcript

Pretemporal transcavernous transtentorial approach for left pontine glioma. A 39-year-old man had worsening headaches, diplopia, dysphagia, and unsteady gait for more than 2 weeks. The MRI showed an intraparenchymal lesion at the left aspect of the pons. The corticospinal tracts were pushed backwards. The tentative diagnosis was a left pontine glioma. We chose a pretemporal transcavernous transtentorial approach to target the lesion. Surgical approaches to the lesions of the ventrolateral brainstem include Dolenc and Kawase approaches. With Dolenc approach, we can access the floor of the third ventricle, the cerebral peduncle, and the upper pons; with Kawase approach, we can target the more lateral aspect of the middle and lower pons. With the combination of both, we have a panoramic view from the midbrain to the pons, which offers a wider area for the selection of suitable entry point to the brainstem. Under general anesthesia, the patient was in supine position with the head fixed by the Mayfield head holder. Intraoperative neuromonitoring was applied. A standard pterional craniotomy was performed with zygoma fracture. Flatten the sphenoid wing. Cut the meningoorbital fold. Expose the periorbita. Peel the lateral wall of the cavernous sinus. Inject tissue glues between V1 and V2. Coagulate and cut the MMA. Expose the GSPN and petrous apex. Expose and remove the ACP extradurally. Expose the third nerve to the oculomotor triangle extradurally. Remove the petrous apex. In this case, the tumor had a diffuse growth from the left cerebral peduncle to the whole left aspect of the pons. In our opinion, if we only used Kawase approach subtemporally, the pontine exposure would be limited, and the traction injury to the dominant temporal lobe was of concern. Hence, the combination of Dolenc and Kawase approaches was adopted. Cut the dura and tentorial incisura along the dashed red line. If the ACP was not removed, mobilization of the third nerve at the oculomotor triangle and the fourth nerve at its entry to the cavernous sinus would be difficult, and the following trans-Meckel’s cave/transtentorial procedures would be impeded as well. The goal of the transcavernous approach is to achieve neurovascular mobilization to gain a wider surgical corridor and to avoid traction injury to the cranial nerves. Cut the tentorium. Open the Meckel’s cave. Ligate the superior petrosal sinus. For a wider surgical exposure, we removed the ACP to access the cerebral peduncle and the upper pons; we cut the tentorial incisura and open the Meckel’s cave to target the middle pons; we removed the petrous apex to go down to the lower pons. This combination offers an immediate proximity to the whole ventrolateral pontine region. Use the neuromonitoring to determine the safe entry point. We tried to preserve the vein running on the surface of the pons. The tumor was removed carefully with the tumor forceps, suction tubes, and bipolar forceps. Debulk the tumor with CUSA. During tumor removal, the MEP and SSEP were constantly monitored. Because the size of the pontine incision was small, we had to remove the tumor from different angles of surgical trajectories. And this trans-Meckel’s cave transtentorial exposure in combination with Dolenc and Kawase approaches sufficed for our need. With the assistance of subcortical mapping, we confined our resection in the tumor, not violating the corticospinal tracts. There was no distinct boundary between the diffuse pontine tumor and the brainstem, so constant subcortical mapping was mandatory. The pathological diagnosis was an anaplastic astrocytoma. This approach offered a wide exposure and a panoramic view to determine the safe entry point to the pons. And it is effective for ventrolateral pontine lesion.

## Time points

0:32 Clinical vignette

0:40 Brain MR

0:53 DTI of corticospinal tracts

0:59 Diagnosis and approach

1:13 3D simulation of the surgical corridor

1:49 Patient positioning

2:01 Operation

9:31 Immediate postop MRI

9:51 Pathology report

9:56 Postop neurological status

10:00 Conclusion

10:09 References
